# Recovering time-varying networks from single-cell data

**DOI:** 10.1093/bioinformatics/btaf210

**Published:** 2025-07-15

**Authors:** Euxhen Hasanaj, Barnabás Póczos, Ziv Bar-Joseph

**Affiliations:** Machine Learning Department, Carnegie Mellon University, Pittsburgh, PA 15213, United States; Machine Learning Department, Carnegie Mellon University, Pittsburgh, PA 15213, United States; Machine Learning Department, Carnegie Mellon University, Pittsburgh, PA 15213, United States; Computational Biology Department, Carnegie Mellon University, Pittsburgh, PA 15213, United States

## Abstract

**Motivation:**

Gene regulation is a dynamic process that underlies all aspects of human development, disease response, and other biological processes. The reconstruction of temporal gene regulatory networks has conventionally relied on regression analysis, graphical models, or other types of relevance networks. With the large increase in time series single-cell data, new approaches are needed to address the unique scale and nature of these data for reconstructing such networks.

**Results:**

Here, we develop a deep neural network, Marlene, to infer dynamic graphs from time series single-cell gene expression data. Marlene constructs directed gene networks using a self-attention mechanism where the weights evolve over time using recurrent units. By employing meta learning, the model is able to recover accurate temporal networks even for rare cell types. In addition, Marlene can identify gene interactions relevant to specific biological responses, including COVID-19 immune response, fibrosis, and aging, paving the way for potential treatments.

**Availability and implementation:**

The code used to train Marlene is available at https://github.com/euxhenh/Marlene.

## 1 Introduction

Biological systems are dynamic, changing over time in response to stimuli and events. To accurately model biological activity during development, disease progression, treatment response, and other processes, it is essential to track how these systems evolve over time (Bar-Joseph *et al.* 2012). Studying the *regulation* of these dynamics is key to understanding the mechanisms that drive them and to identifying interventions that could lead to cures (Silverman *et al.* 2020).

Much of the research in this area focuses on reconstructing regulatory networks (Nguyen *et al.* 2020, Badia-I-Mompel *et al.* 2023). These networks comprise a subset of proteins known as transcription factors (TFs), which regulate the activity of all other genes and proteins within the cell. Most existing approaches represent gene regulatory networks (GRNs) as static graphs. However, even if the underlying interactions are fixed, not all edges are functionally active at every time point (Gasch *et al.* 2000, Luscombe *et al.* 2004). Static models often obscure temporal patterns, making it hard to interpret dynamic processes like disease progression. In contrast, dynamic, edge-activated GRNs, where different subsets of regulatory interactions are active at different times, offer a more accurate representation of gene regulation over time (Karlebach and Shamir 2008, Swift and Coruzzi 2017, Ding *et al.* 2022).

To reconstruct such networks, researchers often integrate static data, such as node types, with dynamic data, such as time series measurements of node activity (gene expression profiles). Early work in this area used microarrays and ChIP-chip data (Lee *et al.* 2002, Wang *et al.* 2006, Gilchrist *et al.* 2009), followed by next-generation time series RNA-seq (Wang *et al.* 2009), and more recently, scRNA-seq (Matsumoto *et al.* 2017, Nguyen *et al.* 2020).

Over the past two decades, several computational methods have been developed to reconstruct dynamic GRNs (Bar-Joseph *et al.* 2003, Schulz *et al.* 2012, Yosef *et al.* 2013, Yan *et al.* 2021). Some rely on time-varying graphical models, including hidden Markov models, Markov random fields, and dynamic Bayesian networks (Ahmed and Xing 2009, Song *et al.* 2009, Dondelinger *et al.* 2013, Zhu and Wang 2015). Others infer temporal precision matrices using variants of the graphical lasso algorithm (Hallac *et al.* 2017, Wang *et al.* 2020).

Although these models have successfully reconstructed certain processes (Ahmed and Xing 2009, Kim *et al.* 2012), they are less suited for newer data types, such as scRNA-seq time series. Traditional graphical models do not scale well to the size of scRNA-seq data. Moreover, they do not explicitly account for the fact that multiple cells and cell types are profiled at each time point. Finally, prior methods do not leverage larger models, such as neural networks, which have shown substantial performance gains across a range of learning tasks (Angermueller *et al.* 2016).

Recently, several methods have used deep learning to infer GRNs (Shu *et al.* 2021, Shrivastava *et al.* 2022). However, few are designed to capture dynamic GRNs directly. Dictys (Wang *et al.* 2023b) is one such method that models dynamics, but relies on data types like ATAC-seq (Buenrostro *et al.* 2015), which provides information about TF binding sites, but is less prevalent and more difficult to obtain.

Outside of biology, dynamic graph inference using neural networks has received growing attention, with applications in areas like information retrieval, molecular graphs, and traffic forecasting (Zhu *et al.* 2021). While these problems share some similarities with dynamic GRN inference, key differences make it hard to apply existing methods directly to time series scRNA-seq data. The problem of inferring temporal graphs typically involves recovering a sequence of adjacency matrices $A_{t}\in R^{n\times n}$ where *n* is the number of nodes, each represented by a *k*-dimensional feature vector. In contrast, with scRNA-seq data, we start with a gene expression matrix $X_{t}\in R^{c\times g}$, where *c* is the number of cells (samples) and *g* is the number of genes (features). The goal is to recover gene networks $A_{t}\in R^{g\times g}$, that is, *graphs of features* rather than nodes (cells).

In this work, we present a deep learning framework that effectively addresses these challenges. Our contributions are three-fold. First, we show that existing deep learning methods for temporal graph structure learning can be adapted to scRNA-seq data analysis. To do this, we introduce a *gene featurization* step using set-based architectures such as DeepSets or Set Transformers (Zaheer *et al.* 2017, Lee *et al.* 2019). Second, we construct dynamic graphs by applying a self-attention mechanism (Bahdanau *et al.* 2014) to these gene feature vectors. To model dynamics, we build on ideas from EvolveGCN, where a gated recurrent unit (GRU) evolves the weights of a graph neural network (Pareja *et al.* 2020). In our approach, however, the GRU evolves the weights of the key and value projection matrices in the self-attention module. This allows for the construction of dynamic graphs that capture regulatory interactions over time. Finally, because GRNs are tied to cell function, separate GRNs must be learned for each cell type. ScRNA-seq datasets often combine multiple cell types, including rare populations. To handle this, we apply model-agnostic meta-learning (MAML) (Finn *et al.* 2017), treating each cell type as a separate “task” to be learned. This allows the model to quickly adapt to cell types with few samples, enabling the reconstruction of dynamic graphs even for rare populations.

**Figure 1.**
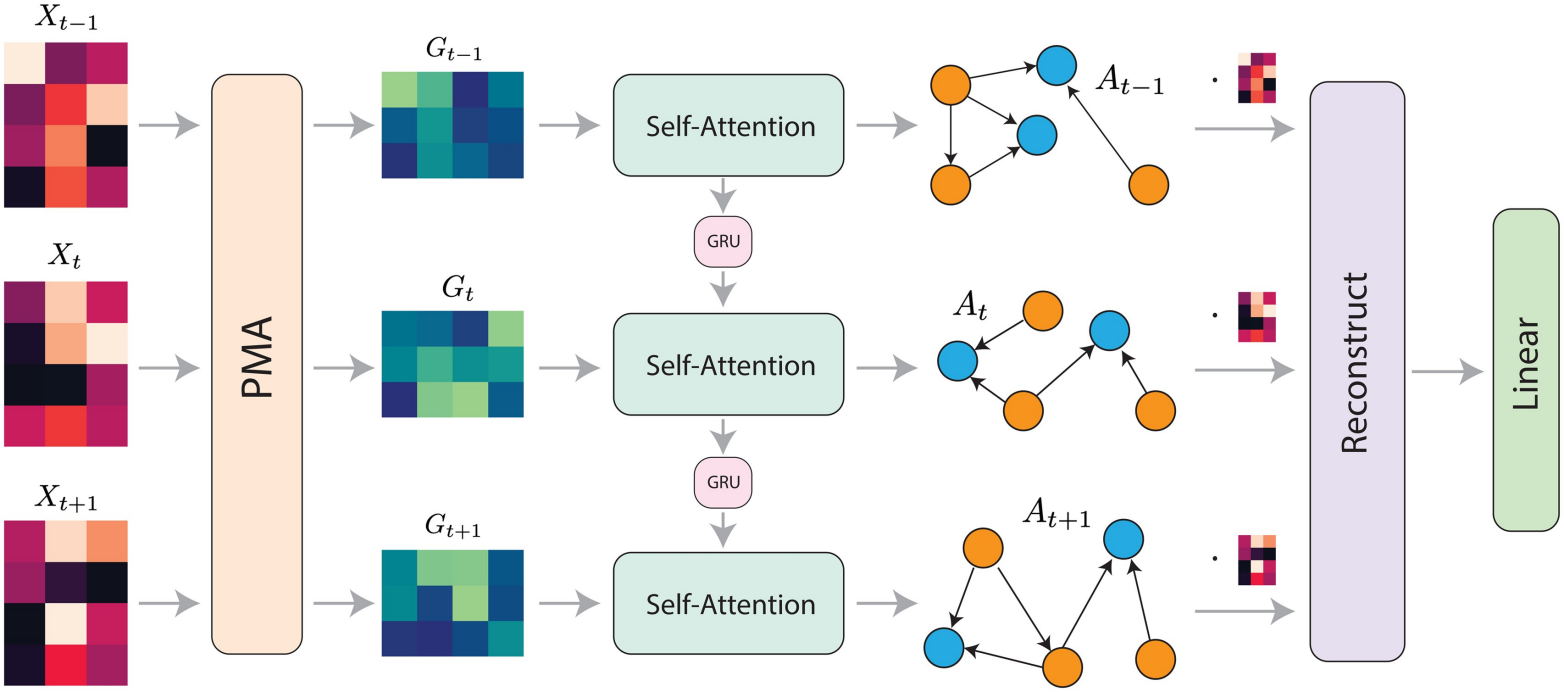
Overview of Marlene. Marlene takes as input a gene expression matrix that is cell-by-gene. It begins by performing gene featurization using a pooling by multihead attention (PMA) mechanism, producing a gene feature matrix. This matrix is then passed through a self-attention module to generate a gene network in the form of an adjacency matrix. To capture temporal dynamics, the weights of the self-attention module evolve across time points via a gated recurrent unit (GRU). The expression of transcription factors, along with the inferred graph, is then used to reconstruct the full gene expression vector. Finally, the reconstructed matrix is used to predict the cell type of each batch. The model is trained using a model-agnostic meta-learning framework, where each cell type is treated as a “task” to be learned, thus allowing fast adaptation to underrepresented cell types.

We apply our *m*et*a* lea*r*ning approach for inferring tempora*l* g*ene* regulatory networks (Marlene, Fig. 1) to three publicly available scRNA-seq datasets. The first is a time series SARS-CoV-2 mRNA vaccination dataset of human peripheral blood mononuclear cells (PBMCs) (Zhang *et al.* 2023). The second is a human lung aging atlas from the Human Cell Atlas Project (Regev *et al.* 2017, Sikkema *et al.* 2023). The third comes from a study of lung fibrosis using a mouse lung injury model (Strunz *et al.* 2020). Each dataset includes multiple time points, enabling longitudinal analysis of biological responses through the inference of dynamic, cell type-specific GRNs. As we show, our method reconstructs accurate networks for these datasets and outperforms existing approaches.

## 2 Methods

### 2.1 Problem setup

Consider a gene expression matrix $X\in R^{c\times g}$, where *c* is the number of cells and *g* is the number of genes. In the dynamic graph setting, we assume each row (cell) is associated with a time point, leading to a time series $\tilde{X}:=\{X_{1},\ldots ,X_{T}\}$, where $X_{t}\in R^{c_{t}\times g}$ and the number of cells *c_t_* may vary with *t*. Our goal is to recover a sequence of directed graphs $\tilde{G}:=\{G_{1},\ldots ,G_{T}\}$, where each $G_{t}=\{N,E_{t}\}$. The node set N=[g] consists of all genes and is assumed to be fixed over time. The dynamic edge sets $E_{t}={\left\{(u,v,w)\right\}}_{u,v\in N,w\in R}$ represent directed, weighted regulatory interactions, where source node *u* regulates target node *v* with strength *w*. Source nodes correspond to TF genes.

Each graph $G_{t}$ can be equivalently represented by an adjacency matrix $A_{t}\in R^{g\times g}$, and we denote the full sequence as $\tilde{A}:=\{A_{1},\ldots ,A_{T}\}$. Because TFs regulate target gene expression, the underlying GRNs should allow reconstruction of the full expression profile of a cell, although other factors including transcription and degradation of mRNA impact expression levels as well. Here, we assume $\tilde{X}=f({\tilde{X}}^{\text{TF}},\tilde{A})$, where ${\tilde{X}}^{\text{TF}}$ is the expression profile of all TFs. The function *f* is unknown as it involves intricate interactions among genes, including combinatorial effects. For example, TFs may act cooperatively to activate a gene, or activation may require the presence of some TFs and absence of others (Wise and Bar-Joseph 2015).

In the deep learning literature, *f* is sometimes modeled using autoencoders (Seninge *et al.* 2021, Shu *et al.* 2021, Wang *et al.* 2023a). However, reconstructing the full gene expression vector is challenging due to extreme sparsity in the data, and standard loss functions like mean squared error tend to overemphasize high-variance or abundant genes. Moreover, full vector reconstruction requires minimizing loss across all genes, including less informative ones (e.g. housekeeping genes). As we later show, predicting cell types, paired with strong regularization to prevent overfitting, offers a more targeted learning objective and leads to more accurate recovery of cell-type specific GRNs. In other words, given a temporal batch of cells of the same type, ${\tilde{X}}^{\text{TF}}$, we frame the problem as a classification task: $y=f({\tilde{x}}^{\text{TF}},\tilde{A})$ where *y* is the known cell type label for the batch. The goal is to learn $\tilde{A}$ from the batch $\tilde{x}$ by minimizing:


(1) $$\text{arg}\min_{\tilde{A}}\text{CrossEntropyLoss}(y,f({\tilde{x}}^{\text{TF}},h(\tilde{x}))),\;\tilde{A}:=h(\tilde{x})$$

for choices of functions *f* and *h*, where *h* maps expression data to adjacency matrices.

### 2.2 Architecture of Marlene

In this work, we propose a neural network architecture called Marlene that effectively learns dynamic GRNs (Fig. 1). Marlene consists of three main steps. The first two define the function *h* in (1), while the third defines the function *f*.

In the first step, we treat each batch of cells as an unordered set and apply a gene featurization operation. The Set Transformer and its predecessor DeepSets are specifically designed to operate on sets, ensuring permutation invariance to the order of samples (Zaheer *et al.* 2017, Lee *et al.* 2019). The Set Transformer uses a pooling by multihead attention (PMA) mechanism to aggregate information from the set. Given an input $X\in R^{c\times g}$, it outputs *k* vectors, forming a matrix $H\in R^{k\times g}$. Each output vector captures a different property of the input. We transpose this to obtain a gene-by-feature matrix $G=H^{⊤}\in R^{g\times k}$ that encodes information about gene activity at each time point. PMA is built on a multihead attention block (MAB) (Vaswani *et al.* 2017), where **X** is the set of keys and the query is a learnable set of *k* vectors $S\in R^{g\times k}$. We use a shared PMA layer across all time points, assuming that the specific statistical properties remain consistent over time (though their value obviously changes for different time points). Given a temporal batch $\tilde{x}=[x_{1},\ldots ,x_{T}]$,


(2) $$\tilde{G}=\text{PMA}{\left(\tilde{x}\right)}^{⊤}:=\text{MAB}{\left(S,\tilde{x}\right)}^{⊤}:={\left(\tilde{M}+\text{rFF}(\tilde{M})\right)}^{⊤}$$

where $M=S+\text{Multihead}(S,x,x)\in R^{g\times k}$ and rFF is a row-wise feedforward layer. Full definitions of these operations are provided in the Supplementary Material.

Note that since PMA aggregates statistics across the input set, mixing cell types within a batch may obscure cell type-specific signals. Therefore, each batch includes cells from a single type only.

In the second step, Marlene constructs a sequence of temporal adjacency matrices using a self-attention mechanism. To model dynamics, we build on EvolveGCN, which uses a GRU to adapt model parameters over time (Pareja *et al.* 2020). While EvolveGCN updates the weights of a graph convolutional layer, we instead evolve the key and query projection weights of the self-attention module using a GRU. As in EvolveGCN, we include a top-*k* pooling step to summarize the gene feature matrix into a square form suitable for the GRU (see Supplementary Material).

More precisely, we initialize the self-attention projection weights $W_{0}^{Q},W_{0}^{K}\in R^{k\times k}$ along with two recurrent units, ${\text{GRU}}_{Q},{\text{GRU}}_{K}$. Given the time sequence of gene feature matrices $\tilde{G}$ from the previous step, we construct the temporal adjacency matrices in a recurrent fashion for all t∈[T]:


(3) $$Z_{t}=\text{TopK}(G_{t})\in R^{k\times k}$$

(4) $$W_{t}^{Q}={\text{GRU}}_{Q}(Z_{t},W_{t-1}^{Q}),\;W_{t}^{K}={\text{GRU}}_{K}(Z_{t},W_{t-1}^{K})$$

(5) $$Q_{t}=G_{t}W_{t}^{Q},\;K_{t}=G_{t}W_{t}^{K}$$

(6) $$A_{t}=\text{softmax}\left(\frac{Q_{t}K_{t}^{⊤}}{\sqrt{k}}\right).$$

Here, $W_{t}^{Q}$ and $W_{t}^{K}$ act as hidden states for their respective GRUs. These GRUs dynamically adapt the self-attention weights over time, guiding which TFs each gene should attend to at each time step. As a result, the evolution of these weights is constrained. To further reduce complexity, we restrict the source nodes (i.e. columns of $A_{t}$) to a set of *p* known TFs from the TRRUST database (Han *et al.* 2018), which reduces the number of learnable parameters. In our implementation, this results in $A_{t}\in R^{g\times p}$.

Next, we reconstruct gene expression based on the TF expression and the inferred adjacency matrices. This is then passed through one or more fully connected layers with nonlinear activation functions *σ*. Finally, we sum across the output vectors to produce a logit vector with the same dimension as the number of cell types in the data:


(7) $$\tilde{y}=\text{Pool}(\text{Linear}(\ldots \sigma (\text{Linear}({\tilde{x}}^{\text{TF}}{\tilde{A}}^{⊤})))).$$

The depth of the neural network can be increased by stacking MAB layers during gene featurization, stacking GRUs, or stacking more linear layers at the end.

### 2.3 Meta learning for rare cell types

ScRNA-seq datasets often originate from heterogeneous samples, potentially containing multiple distinct cell types. Some of these cell subpopulations are rare and represented by only a small number of cells. Since our goal is to recover cell type-specific temporal GRNs, learning large graphs for these rare cell types may not be feasible and be prone to overfitting. Since many interactions are shared across cell types (Chasman and Roy 2017), we employ the MAML framework (Finn *et al.* 2017). MAML is designed to help neural networks adapt to novel tasks with limited training samples (i.e. few shot learning). By treating each cell type as a “task,” the MAML training paradigm facilitates the recovery of dynamic graphs for rare cell types. The training loop begins by adapting model parameters using several gradient steps on a batch of support cells. These adapted parameters are then evaluated on a separate set of query cells, followed by a meta-update.

### 2.4 Ground truth databases used for benchmarking

For the human genome, TRRUST contains 8427 unique validated regulatory edges, while RegNetwork includes 150 405. Some edges were excluded from the analysis due to missing TFs or target genes in the expression data. We used only genes present in TRRUST. For the mouse lung dataset, we used the corresponding mouse databases. To approximate the number of links in these databases, we selected the top 2% of predicted edges from each method. For Marlene, this was done by sparsifying the self-attention matrix to retain only the top scoring edges. Overlap significance was assessed using Fisher’s exact test (Fisher 1922), and all *P*-values were corrected for multiple testing using the Benjamini–Hochberg procedure (Benjamini and Hochberg 1995).

### 2.5 Baselines

We compare Marlene against several popular static gene regulatory network inference methods from the BEELINE benchmark (Pratapa *et al.* 2020) and beyond, including PIDC, GENIE3, GRNBoost2, SCODE, and DeepSEM (Huynh-Thu *et al.* 2010, Chan *et al.* 2017, Matsumoto *et al.* 2017, Moerman *et al.* 2019, Shu *et al.* 2021). These methods are applied independently at each time point. PIDC uses partial information decomposition to assess gene–gene relationships. GENIE3 and GRNBoost2 are based on decision trees and gradient boosting, respectively. SCODE models gene dynamics using ordinary differential equations, while DeepSEM is a deep generative model grounded in structural equation modeling. We also compare against time-varying graphical lasso (TVGL) (we used the implementation of https://github.com/fdtomasi/regain), which models temporal precision matrices (Hallac *et al.* 2017), and against a deep neural network that utilizes the S4 module (GraphS4mer) (Gu *et al.* 2021, Tang *et al.* 2022).

### 2.6 Training procedure and selection of hyperparameters

During inference, we obtain multiple adjacency matrices *A_t_* across different batches and average them to produce the final GRN. Marlene is trained with a batch size of 16 cells and uses 16 learnable seed vectors in the PMA layer. For MAML, we use five inner loops. The model checkpoint with the lowest loss is selected for GRN inference.

We used standard gradient descent for the inner update, while for the meta-update, we employ Adam (Kingma and Ba 2014). Gradient clipping is critical during training to prevent overfitting to the batch cell type. For the meta-update, we use a decaying learning rate starting at 1e−4, and for the inner loop, we use a fixed learning rate of 1e‐3 across datasets. All experiments were run on an NVIDIA RTX 3060 GPU and completed within a few minutes per run.

## 3 Results

In this work, we introduce Marlene, an attention-based neural network for inferring temporal GRNs from scRNA-seq data. Marlene first extracts gene features using a set-based operation from Set Transformers. It then performs model adaptation via a GRU, which evolves the projection matrices of a self-attention layer. Self-attention is used to generate dynamic adjacency matrices, representing these temporal GRNs.

To validate our approach, we analyze three publicly available scRNA-seq datasets (Table 1): a human SARS-CoV-2 mRNA vaccination dataset, a lung aging atlas (the Human Lung Cell Atlas—HLCA), and a mouse lung fibrosis dataset (Strunz *et al.* 2020, Sikkema *et al.* 2023, Zhang *et al.* 2023). We assess the quality of the inferred networks using two literature-curated databases of TF-gene interactions: TRRUST and RegNetwork (Liu *et al.* 2015, Han *et al.* 2018). Finally, we benchmark Marlene against several popular baselines.

**Table 1.** Time series scRNA-seq datasets used in this study.

				Number of		Metadata
Dataset	Cells	Genes^a^	TFs	Cell types	Time points	Sample
SARS-CoV-2	113 271	1899	556	7	d0, d2, d10, d28	PBMCs (human)
HLCA	27 953^b^	2433	674	11	Ages <35, 35–50, ≥50	Lung (human)
Fibrosis	22 758	1217	433	6	PBS, d3, d7, d10, d14, d21, d28	Lung (mouse)

Preprocessing information are shown in the Supplementary Material.

a Only showing the number of genes overlapping with the TRRUST database.

b We randomly sampled cells from 11 cell types.

### 3.1 Case study 1: SARS-CoV-2 vaccination

The SARS-CoV-2 vaccination dataset contains PBMCs from six healthy donors, sampled at four time points: days 0, 2, 10, and 28 (Zhang *et al.* 2023). Day 0 samples represent pre-vaccination baseline. We removed the “Other” cell type group and kept the remaining seven, including B cells, dendritic cells, monocytes (Mono), natural killer cells (NK), and various T cell subtypes.

#### 3.1.1 Marlene recovers accurate gene regulatory networks

**Figure 2.**
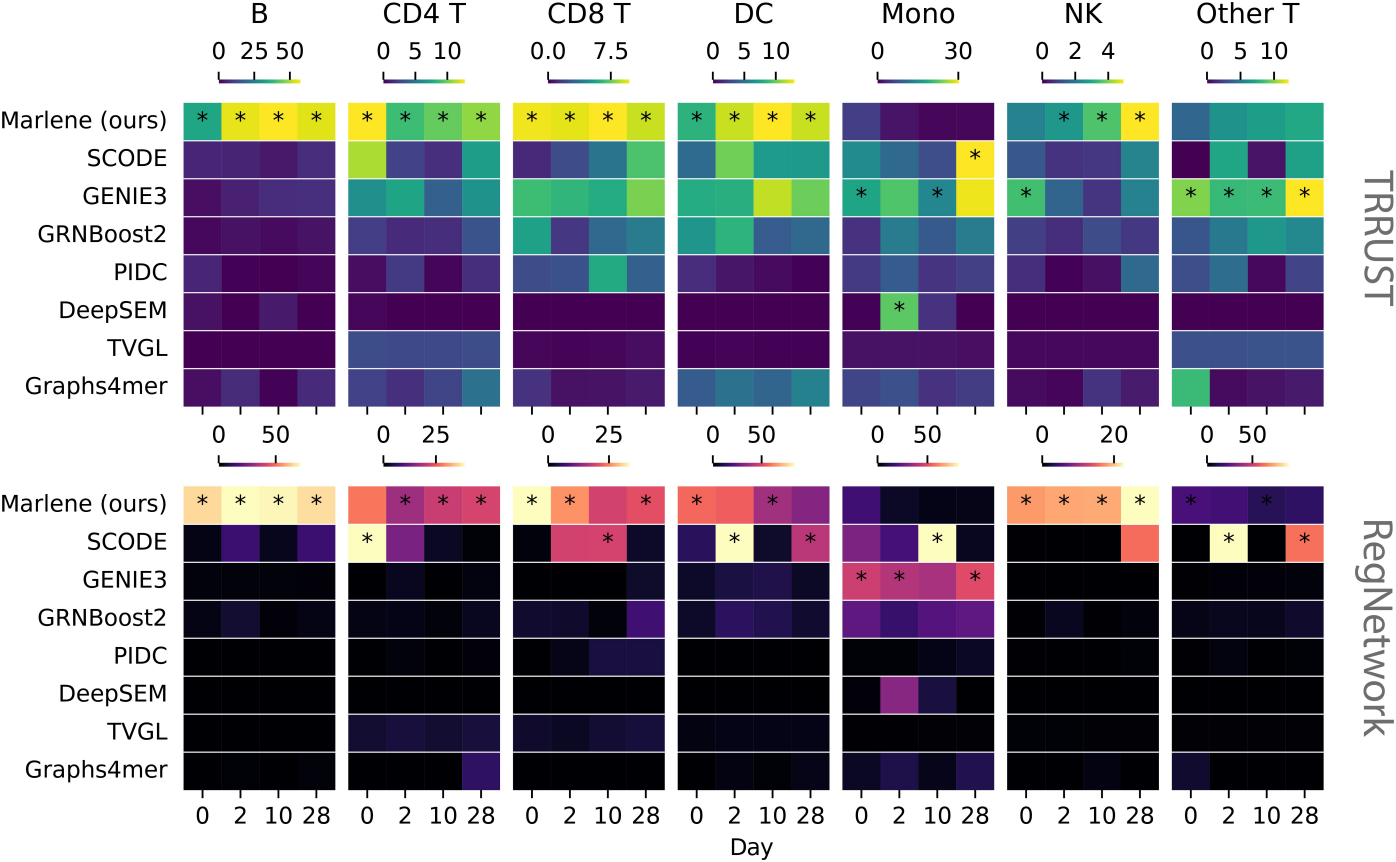
Overlap analysis of the SARS-CoV-2 dataset. Showing $-{\;\text{log}\;}_{10}(\text{FDR})$ values from a Fisher’s exact test measuring the overlap between predicted TF-gene interactions in reconstructed networks and two TF-gene interaction databases, TRRUST (top) and RegNetwork (bottom). Each column block is a cell type. Best performing method is starred. Exact values shown in Supplementary Fig. S1.

Analysis results using Marlene and prior methods are presented in Fig. 2. As shown, Marlene outperformed competing approaches in GRN inference for 5 of the 7 cell types, yielding statistically significant results across time points. For example, for B cells, Marlene identified over 800 regulatory links from RegNetwork at each time point (FDR ≤1e‐67), outperforming the next best method, SCODE, which detected 579 links on day 2 (FDR ≤1e‐15). Similar patterns were observed for NK cells, where Marlene identified over 600 RegNetwork links per time point (FDR ≤1e‐18), while SCODE showed significant overlap at only one time point (day 28). Using the TRRUST database as a reference, performance differences were less pronounced. Nonetheless, Marlene achieved higher overlap for 5 of the 7 cell types, followed by GENIE3, which performed best for monocytes and the “Other T” category.

We also calculated the pairwise overlap of predicted edges across cell types for Marlene. As can be seen, while some overlap exists, our method learns cell type specific interaction networks. As expected, the highest overlap was observed among the three T-cell subtypes, while other myeloid-derived populations such as monocytes and dendritic cells showed lower overlap with T cells (Supplementary Fig. S2a).

#### 3.1.2 Marlene recovers realistic dynamic transitions

**Figure 3.**
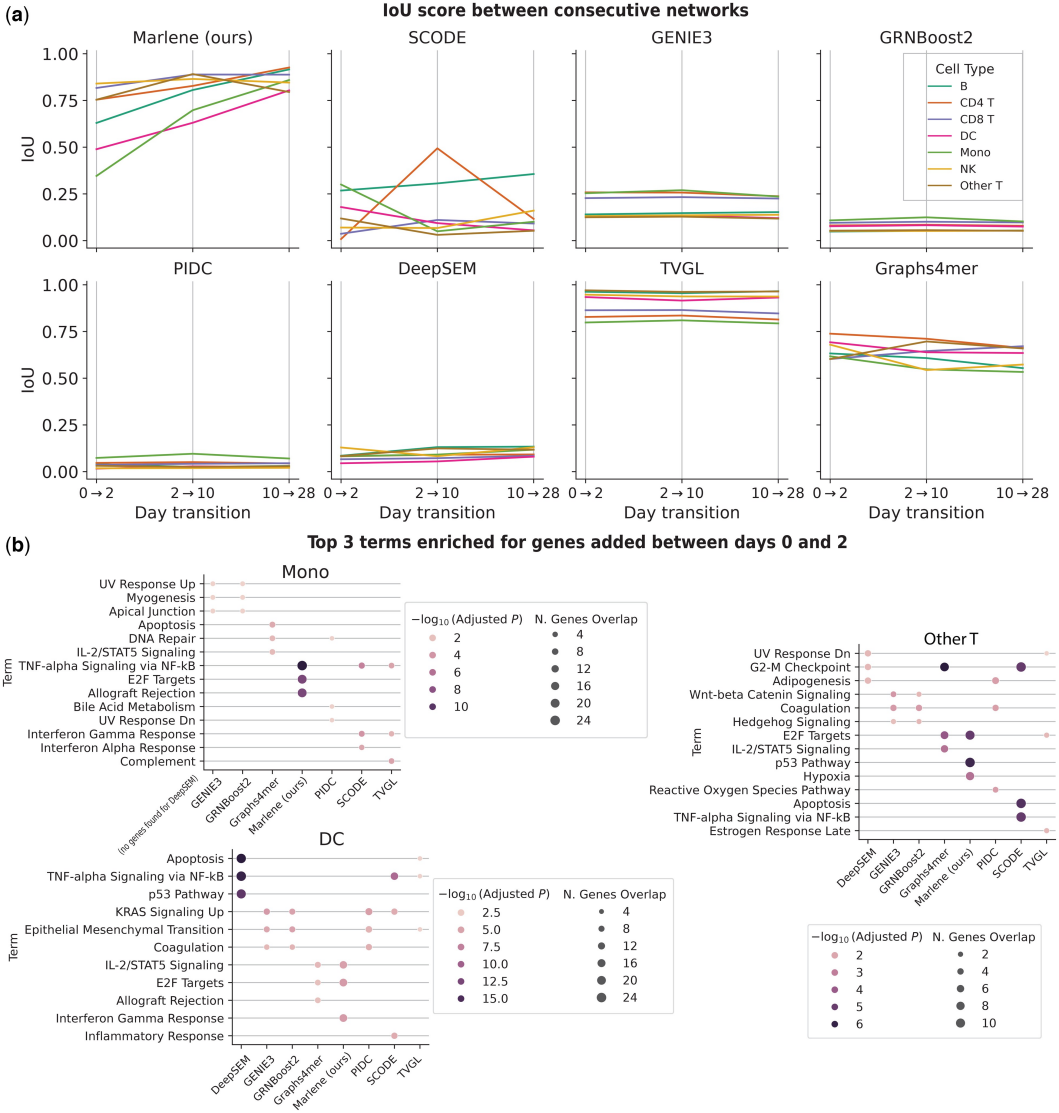
Temporal analysis of the predicted gene regulatory networks for the SARS-CoV-2 vaccine dataset. (a) Intersection-over-union (IoU) scores between consecutive graphs. (b) For each method, top 3 MSigDB terms enriched for genes that were newly regulated at day 2 but not day 0.

So far, our analysis has focused on individual time points. We next assessed the quality of *transitions* between inferred graphs at consecutive time points—specifically, whether the learned graphs evolved smoothly over time. To quantify this, we computed the intersection-over-union (IoU) score between edge sets at time points *t* and *t* + 1 (Fig. 3a). For most cell types, Marlene showed the lowest IoU score during the initial transition (days 0→2), followed by higher scores between days 2→10, and 10→28. This aligns with biological expectations, as shifts in gene expression are likely to be most pronounced shortly after vaccination. Among the baselines, GENIE3, GRNBoost2, SCODE, PIDC, and DeepSEM exhibited consistently low IoU scores across all transitions, likely due to their lack of temporal modeling. TVGL, in contrast, produced uniformly high IoU scores that changed little over time. Graphs4mer showed a gradual decline in IoU scores across time, which contradicts the expected spike in regulation following vaccination.

Next, we evaluated the biological relevance of TF-gene regulatory links added between time points, focusing on the initial transition (days 0→2), as this period is likely to witness a more significant immune response. For each cell type, we identified genes newly regulated by some TF at day 2 but not at day 0. Using this set of genes *z*, we performed gene set enrichment analysis (GSEA) (Subramanian *et al.* 2005, Fang *et al.* 2023) with the MSigDB gene set collection (Liberzon *et al.* 2015). GSEA assigns an enrichment score to *z* reflecting its overrepresentation within the curated gene sets. We found that for many cell types, genes newly regulated at day 2 by Marlene showed strong enrichment for COVID-19 and SARS-CoV-2 related pathways. For example, the “Interferon Gamma Response,” which was identified as a SARS-CoV-2 antiviral response (Hilligan *et al.* 2023), was significantly enriched in dendritic cells (15 genes, FDR =1e‐6). Similarly, “TNF-alpha Signaling via NF-kB”—a pathway central to inflammation and immune response (Hayden and Ghosh 2011)—appeared in multiple cell types, along with stress-related processes such as “Apoptosis” and the “p53 Pathway” (Harris and Levine 2005, Elmore 2007). Other methods showed some enrichment for relevant terms but had smaller gene overlaps for these types (Fig. 3b) or less consistency across cell types (e.g. DeepSEM, SCODE).

Overall, these results suggest that Marlene is able to capture both known TF-gene links, but also genes that are relevant to the response being studied.

#### 3.1.3 Ablation study

To assess the contribution of Marlene’s components, we ran two additional experiments. First, in an ablation study, we tested a version of Marlene without the GRU (time-independent networks), and a version where the PMA layer was replaced with a DeepSet layer. In both cases, Marlene outperformed the ablated variants on both ground truth databases (Supplementary Fig. S2b). In a second experiment, we modified the training objective by having Marlene predict the gene expression vector instead of the cell type (Marlene GEX). Again, Marlene outperformed this variant across all cell types (Supplementary Fig. S2c).

### 3.2 Case study 2: aging and senescence in the lung

The HLCA is a large data integration effort by the Human Cell Atlas Project (Regev *et al.* 2017, Sikkema *et al.* 2023). These data combine scRNA-seq samples from 107 individuals spanning an age range of 10–76 years, making it particularly valuable for studying aging and senescence (a form of aging characterized by the absence of cell division) (van Deursen 2014, SenNet Consortium 2022).

We split the atlas into three age groups at 35 and 50 years old, thus forming a pseudotime series of length 3. We removed smokers from the dataset as these will likely confound the results. To accommodate the data in the GPU, we randomly selected cells from 11 cell types, including Type II pneumocytes, endothelial cells, and monocytes. Additional analysis, in which we tested the impact of changing the number of cell types we model, is presented in Supplementary Fig. S4c.

Similar to the vaccination dataset, we begin the analysis by evaluating the set of regulatory links using the TRRUST and RegNetwork databases. For this dataset, we find that Marlene and SCODE are the top two performing methods (Supplementary Figs S3a and 4a). For some of the cell types, Marlene achieves significant results, recovering more than 1000 RegNetwork links (classical monocytes, FDR =1e‐76). Even for cell types with fewer cells, such as nonclassical monocytes (with only 138 cells for the second age group), Marlene still recovered more than 800 known TF-gene links for each transition (FDR ≤1e‐27). SCODE performed well for some cell types such as CD1c-positive myeloid dendritic cells and CD4-positive, alpha-beta T cells. For all other methods, the overlap was smaller. Note that while SCODE is comparable for the static network (single time point) inference task, it does not utilize dynamic information.

We next examined the ability of different methods to capture the dynamics of the biological processes. For this, we looked at graph transitions. IoU scores show that only the temporal methods (Marlene, TVGL, Graphs4mer) capture the smooth temporal transition between time points, while other methods, including SCODE, achieve low IoU scores (Supplementary Fig. S4b). We performed GSEA using Jensen Diseases gene set to see if genes added by Marlene in these transitions were enriched for any age-related diseases (Pletscher-Frankild *et al.* 2015, Grissa *et al.* 2022). We found that Marlene added genes that are enriched for several diseases such as arthritis, lung disease, and coronary artery disease. Other dynamic baselines were also enriched for relevant terms, but contained fewer marker genes (Supplementary Fig. S3b).

**Figure 4.**
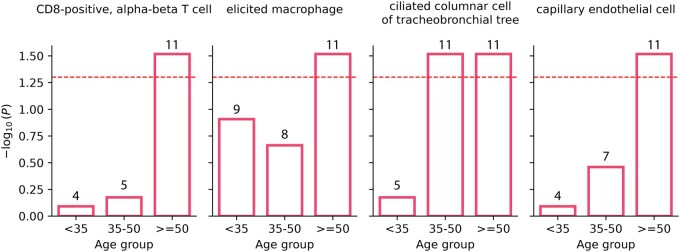
Enrichment for senescence using the SenMayo set. For four cell types, there was statistically significant enrichment for the oldest age group. We only used the top 200 regulated genes.

We also investigated whether genes regulated in different age groups were enriched for senescence. Cellular senescence refers to a permanent arrest of cell division triggered by accumulation of DNA damage (Suryadevara *et al.* 2024). The absence of cell division can negatively impact tissue regeneration and repair, thus contributing to various age-related diseases. Here, we use the SenMayo gene set which contains 125 genes reported to be enriched for senescence (Saul *et al.* 2022). Only 81 of these genes overlapped with our data. We found that for 4 cell types, there was an increase in SenMayo gene regulation in the oldest age group (age > 50), suggesting that senescent cells accumulate with age as hypothesized (Fig. 4).

### 3.3 Case study 3: fibrosis in a mouse lung injury model

Next, we evaluated whether Marlene could perform effectively across different species by analyzing a dataset from a mouse model of lung injury induced by the chemotherapeutic agent bleomycin (Strunz *et al.* 2020). The dataset included seven time points: one pretreatment and six posttreatment intervals. After filtering out cell types with low representation and genes with low counts, we retained six cell types, including B cells, T cells, and macrophages.

In this analysis, Marlene outperformed competing methods in 4 of 6 cell types when benchmarked against RegNetwork, including alveolar epithelial cells, dendritic cells, endothelial cells, and macrophages. For T cells, TVGL showed slightly better performance. When evaluated against the TRRUST database, SCODE performed well in four cell types, while Marlene surpassed it in the remaining two. The differing results between two databases may reflect their incomplete coverage, highlighting the need for further refinement.

Finally, all static baselines, including SCODE, showed low IoU scores across time points, indicating their inability to capture temporal evolution. In contrast, Marlene, showed increasing IoU scores over time, suggesting ongoing lung regeneration which slowly stabilizes (Supplementary Fig. S5).

## 4 Discussion

Gene regulation is a dynamic process that underlies all biological systems. Understanding which TFs regulate which genes, and when this regulation occurs, provides insights into these dynamic processes which can lead to better treatment options. For instance, understanding what TF-gene links are disrupted could help researchers discover drugs targets for specific TF-gene connections.

To improve on current methods for reconstructing time varying regulatory networks, we use the expressive capabilities of deep neural networks to model the dynamic regulation of genes. Specifically, we focus on inferring dynamic networks from scRNA-seq data.

Our proposed method, Marlene, constructs dynamic graphs from time series data. Marlene begins with a set pooling operator based on PMA to extract gene features. These gene features are then used to construct dynamic graphs via a self-attention mechanism. The weights of the self-attention block are updated through the use of GRUs. Furthermore, employing MAML, we help Marlene uncover graphs even for rare cell types. However, Marlene optimizes the prediction of cell type label rather than gene expression. As such, Marlene is not currently equipped to determine the impact of perturbations including gene knockouts or overexpression experiments. Exploring the integration of causal inference capabilities into Marlene represents a promising direction for future research.

We demonstrated the effectiveness of Marlene in recovering dynamic GRNs using three datasets: a SARS-CoV-2 vaccination dataset, a lung aging atlas, and a mouse dataset of fibrosis. In all three datasets, Marlene successfully identified many validated TF-gene links from the TRRUST and RegNetwork databases across various cell types. It also accurately modeled the temporal dynamics of these connections. Prior methods that ignore temporal continuity often lead to disjoint networks between consecutive time points. Other methods combined all time points together leading to very similar networks across all time points. In contrast, Marlene accurately recovered the variation dynamics, which is often characterized by strong rewiring following treatment that later stabilizes. In addition, Marlene identified many relevant edges. For instance, in the lung aging data, several dynamic edges were enriched for age-related diseases, such as arthritis. Meanwhile, in the SARS-CoV-2 data, these dynamic links were enriched for immune response processes. Prior methods captured some known edges, however, the overall results were less significant. By providing better models to explain disease and vaccine response, researchers can zoom in on the specific mechanisms targeted which in turn can lead to better treatments.

## 5 Limitations and future work

While effective, Marlene has a few limitations. First, it relies solely on scRNA-seq data and does not integrate additional modalities. Complementary data sources, such as ATAC-seq, could better constrain the space of regulatory interactions and improve model identifiability. Furthermore, the datasets we used in this study, while typical for scRNA-seq time series, consisted of only a few time points. For longer sequences, the GRU operation may suffer from vanishing gradient problems (Pascanu *et al.* 2012). In such scenarios, the S4 module may be preferred as it has been shown to model long sequences better than traditional GRUs (Gu *et al.* 2021). In addition, using a large number of genes for training, results in quadratic growth in memory consumption due to the need to store adjacency matrices. This led us to restrict the set of genes for each of the two studies. A more efficient implementation or alternative approaches such as FlashAttention (Dao *et al.* 2022) can lead to better ability to utilize all genes profiled.

## Supplementary Material

btaf210_Supplementary_Data

## Data Availability

The datasets were sourced from thier respective papers: SARS-CoV-2 (https://zenodo.org/records/7555405), HLCA (https://data.humancellatlas.org/hca-bio-networks/lung/atlases/lung-v1-0), Fibrosis (GSE141259).

## References

[btaf210-B1] Ahmed A , Xing EP. Recovering time-varying networks of dependencies in social and biological studies. Proc Natl Acad Sci U S A 2009;106:11878–83. 19570995 10.1073/pnas.0901910106 PMC2704856

[btaf210-B2] Angermueller C , Pärnamaa T, Parts L et al Deep learning for computational biology. Mol Syst Biol 2016;12:878. 27474269 10.15252/msb.20156651 PMC4965871

[btaf210-B3] Badia-I-Mompel P , Wessels L, Müller-Dott S et al Gene regulatory network inference in the era of single-cell multi-omics. Nat Rev Genet 2023;24:739–54. 37365273 10.1038/s41576-023-00618-5

[btaf210-B4] Bahdanau D , Cho K, Bengio Y. Neural machine translation by jointly learning to align and translate. In: International Conference on Learning Representations, 2015.

[btaf210-B5] Bar-Joseph Z , Gerber GK, Lee TI et al Computational discovery of gene modules and regulatory networks. Nat Biotechnol 2003;21:1337–42. 14555958 10.1038/nbt890

[btaf210-B6] Bar-Joseph Z , Gitter A, Simon I. Studying and modelling dynamic biological processes using time-series gene expression data. Nat Rev Genet 2012;13:552–64. 22805708 10.1038/nrg3244

[btaf210-B7] Benjamini Y , Hochberg Y. Controlling the false discovery rate: a practical and powerful approach to multiple testing. J R Statist Soc Ser B Methodol 1995;57:289–300.

[btaf210-B8] Buenrostro JD , Wu B, Chang HY et al ATAC-seq: a method for assaying chromatin accessibility genome-wide. Curr Protoc Mol Biol 2015;109:21.29.1–9. 10.1002/0471142727.mb2129s109 PMC4374986 25559105

[btaf210-B9] Chan TE , Stumpf MPH, Babtie AC. Gene regulatory network inference from single-cell data using multivariate information measures. Cell Syst 2017;5:251–67.e3. 28957658 10.1016/j.cels.2017.08.014 PMC5624513

[btaf210-B10] Chasman D , Roy S. Inference of cell type specific regulatory networks on mammalian lineages. Curr Opin Syst Biol 2017;2:130–9. 29082337 10.1016/j.coisb.2017.04.001 PMC5656272

[btaf210-B11] Dao T et al FlashAttention: fast and memory—efficient exact attention with IO-awareness. In: International Conference on Neural Information Processing Systems, 2022, 16344–59.

[btaf210-B12] Ding J , Sharon N, Bar-Joseph Z. Temporal modelling using single-cell transcriptomics. Nat Rev Genet 2022;23:355–68. 35102309 10.1038/s41576-021-00444-7 PMC10354343

[btaf210-B13] Dondelinger F , Lèbre S, Husmeier D. Non-homogeneous dynamic Bayesian networks with Bayesian regularization for inferring gene regulatory networks with gradually time-varying structure. Mach Learn 2013;90:191–230.

[btaf210-B14] Elmore S. Apoptosis: a review of programmed cell death. Toxicol Pathol 2007;35:495–516. 17562483 10.1080/01926230701320337 PMC2117903

[btaf210-B15] Fang Z , Liu X, Peltz G. GSEApy: a comprehensive package for performing gene set enrichment analysis in python. Bioinformatics 2023;39:btac757. 10.1093/bioinformatics/btac757 PMC9805564 36426870

[btaf210-B16] Finn C , Abbeel P, Levine S. Model-agnostic meta-learning for fast adaptation of deep networks. *Int Conf Mach Learn* 2017;**70**:1126–1135.

[btaf210-B17] Fisher RA. On the interpretation of $\chi^{2}$ from contingency tables, and the calculation of P. J R Stat Soc 1922;85:87.

[btaf210-B18] Gasch AP, Spellman PT, Kao CM et al Genomic expression programs in the response of yeast cells to environmental changes. Mol Biol Cell 2000;11:4241–57. 11102521 10.1091/mbc.11.12.4241 PMC15070

[btaf210-B19] Gilchrist DA , Fargo DC, Adelman K. Using ChIP-chip and ChIP-seq to study the regulation of gene expression: genome-wide localization studies reveal widespread regulation of transcription elongation. Methods 2009;48:398–408. 19275938 10.1016/j.ymeth.2009.02.024 PMC3431615

[btaf210-B20] Grissa D , Junge A, Oprea TI et al Diseases 2.0: a weekly updated database of disease-gene associations from text mining and data integration. Database (Oxford) 2022;2022:baac019. 35348648 10.1093/database/baac019 PMC9216524

[btaf210-B21] Gu A , Goel K, Ré C. Efficiently modeling long sequences with structured statespaces. In: International Conference on Learning Representations, 2022.

[btaf210-B22] Hallac D , Park Y, Boyd S et al Network inference via the time-varying graphical lasso. In: *KDD*. New York, NY, USA: Association for Computing Machinery, 2017, 205–13. 10.1145/3097983.3098037 PMC5951186 29770256

[btaf210-B23] Han H , Cho J-W, Lee S et al TRRUST v2: an expanded reference database of human and mouse transcriptional regulatory interactions. Nucleic Acids Res 2018;46:D380–6. 29087512 10.1093/nar/gkx1013 PMC5753191

[btaf210-B24] Harris SL , Levine AJ. The p53 pathway: positive and negative feedback loops. Oncogene 2005;24:2899–908. 15838523 10.1038/sj.onc.1208615

[btaf210-B25] Hayden MS , Ghosh S. NF-kb in immunobiology. Cell Res 2011;21:223–44. 21243012 10.1038/cr.2011.13 PMC3193440

[btaf210-B26] Hilligan KL , Namasivayam S, Clancy CS et al Bacterial-induced or passively administered interferon gamma conditions the lung for early control of SARS-CoV-2. Nat Commun 2023;14:8229. 38086794 10.1038/s41467-023-43447-0 PMC10716133

[btaf210-B27] Huynh-Thu VA , Irrthum A, Wehenkel L et al Inferring regulatory networks from expression data using tree-based methods. PLoS One 2010;5:e12776. 20927193 10.1371/journal.pone.0012776 PMC2946910

[btaf210-B28] Karlebach G , Shamir R. Modelling and analysis of gene regulatory networks. Nat Rev Mol Cell Biol 2008;9:770–80. 18797474 10.1038/nrm2503

[btaf210-B29] Kim M-S , Kim J-R, Kim D et al Spatiotemporal network motif reveals the biological traits of developmental gene regulatory networks in *Drosophila melanogaster*. BMC Syst Biol 2012;6:31. 22548745 10.1186/1752-0509-6-31 PMC3434043

[btaf210-B30] Kingma DP , Ba J. Adam: a method for stochastic optimization. In: *International Conference on Learning Representations*, 2015.

[btaf210-B31] Lee J, Lee Y, Kim J et al Set transformer: a framework for attention-based permutation-invariant neural networks. In: *Proceedings of the 36th International Conference on Machine Learning*, Long Beach, California, Vol. 97, p.3744–53. PMLR, 2019.

[btaf210-B32] Lee TI , Rinaldi NJ, Robert F et al Transcriptional regulatory networks in *Saccharomyces cerevisiae*. Science 2002;298:799–804. 12399584 10.1126/science.1075090

[btaf210-B33] Liberzon A , Birger C, Thorvaldsdóttir H et al The molecular signatures database (MSigDB) hallmark gene set collection. Cell Syst 2015;1:417–25. 26771021 10.1016/j.cels.2015.12.004 PMC4707969

[btaf210-B34] Liu Z-P , Wu C, Miao H et al RegNetwork: an integrated database of transcriptional and post-transcriptional regulatory networks in human and mouse. Database (Oxford) 2015;2015:bav095. 26424082 10.1093/database/bav095 PMC4589691

[btaf210-B35] Luscombe NM , Babu MM, Yu H et al Genomic analysis of regulatory network dynamics reveals large topological changes. Nature 2004;431:308–12. 15372033 10.1038/nature02782

[btaf210-B36] Matsumoto H , Kiryu H, Furusawa C et al SCODE: an efficient regulatory network inference algorithm from single-cell RNA-seq during differentiation. Bioinformatics 2017;33:2314–21. 28379368 10.1093/bioinformatics/btx194 PMC5860123

[btaf210-B37] Moerman T , Aibar Santos S, Bravo González-Blas C et al GRNBoost2 and arboreto: efficient and scalable inference of gene regulatory networks. Bioinformatics 2019;35:2159–61. 30445495 10.1093/bioinformatics/bty916

[btaf210-B38] Nguyen H , Tran D, Tran B et al A comprehensive survey of regulatory network inference methods using single-cell RNA sequencing data. Brief Bioinform 2020;22:bbaa190. 10.1093/bib/bbaa190 PMC8138892 34020546

[btaf210-B39] Pareja A , Domeniconi G, Chen J et al EvolveGCN: evolving graph convolutional networks for dynamic graphs. Proc AAAI Conf Artif Intell 2020;34:5363–70.

[btaf210-B40] Pascanu R , Mikolov T, Bengio Y. On the difficulty of training recurrent neural networks. In: *Proceedings of the 30th International Conference on Machine Learning*, PMLR 2013;**28**:1310–18.

[btaf210-B41] Pletscher-Frankild S , Pallejà A, Tsafou K et al DISEASES: text mining and data integration of disease-gene associations. Methods 2015;74:83–9. 25484339 10.1016/j.ymeth.2014.11.020

[btaf210-B42] Pratapa A , Jalihal AP, Law JN et al Benchmarking algorithms for gene regulatory network inference from single-cell transcriptomic data. Nat Methods 2020;17:147–54. 31907445 10.1038/s41592-019-0690-6 PMC7098173

[btaf210-B43] Regev A , Teichmann SA, Lander ES et al; Human Cell Atlas Meeting Participants. The human cell atlas. Elife 2017;6:e27041. 10.7554/eLife.27041 PMC5762154 29206104

[btaf210-B44] Saul D , Kosinsky RL, Atkinson EJ et al A new gene set identifies senescent cells and predicts senescence-associated pathways across tissues. Nat Commun 2022;13:4827. 35974106 10.1038/s41467-022-32552-1 PMC9381717

[btaf210-B45] Schulz MH , Devanny WE, Gitter A et al DREM 2.0: improved reconstruction of dynamic regulatory networks from time-series expression data. BMC Syst Biol 2012;6:104. 22897824 10.1186/1752-0509-6-104 PMC3464930

[btaf210-B46] Seninge L , Anastopoulos I, Ding H et al VEGA is an interpretable generative model for inferring biological network activity in single-cell transcriptomics. Nat Commun 2021;12:5684. 34584103 10.1038/s41467-021-26017-0 PMC8478947

[btaf210-B47] SenNet Consortium. NIH SenNet Consortium to map senescent cells throughout the human lifespan to understand physiological health. Nat Aging 2022;2:1090–100. 36936385 10.1038/s43587-022-00326-5 PMC10019484

[btaf210-B48] Shrivastava H , Zhang X, Song L et al GRNUlar: a deep learning framework for recovering single-cell gene regulatory networks. J Comput Biol 2022;29:27–44. 35050715 10.1089/cmb.2021.0437

[btaf210-B49] Shu H , Zhou J, Lian Q et al Modeling gene regulatory networks using neural network architectures. Nat Comput Sci 2021;1:491–501. 38217125 10.1038/s43588-021-00099-8

[btaf210-B50] Sikkema L , Ramírez-Suástegui C, Strobl DC et al; Lung Biological Network Consortium. An integrated cell atlas of the lung in health and disease. Nat Med 2023;29:1563–77. 37291214 10.1038/s41591-023-02327-2 PMC10287567

[btaf210-B51] Silverman EK et al Molecular networks in network medicine: development and applications. Wiley Interdiscip Rev Syst Biol Med 2020;12:e1489. 32307915 10.1002/wsbm.1489 PMC7955589

[btaf210-B52] Song L , Kolar M, Xing E. Time-varying dynamic Bayesian networks. In: *Advances in Neural Information Processing Systems*, Vancouver, B.C., Canada, 2009, 22.

[btaf210-B53] Strunz M , Simon LM, Ansari M et al Alveolar regeneration through a Krt8+ transitional stem cell state that persists in human lung fibrosis. Nat Commun 2020;11:3559. 32678092 10.1038/s41467-020-17358-3 PMC7366678

[btaf210-B54] Subramanian A , Tamayo P, Mootha VK et al Gene set enrichment analysis: a knowledge-based approach for interpreting genome-wide expression profiles. Proc Natl Acad Sci U S A 2005;102:15545–50. 16199517 10.1073/pnas.0506580102 PMC1239896

[btaf210-B55] Suryadevara V, Hudgins AD, Rajesh A et al SenNet recommendations for detecting senescent cells in different tissues. Nat Rev Mol Cell Biol 2024;1–23. 38831121 10.1038/s41580-024-00738-8 PMC11578798

[btaf210-B56] Swift J , Coruzzi GM. A matter of time—how transient transcription factor interactions create dynamic gene regulatory networks. Biochim Biophys Acta Gene Regul Mech 2017;1860:75–83. 27546191 10.1016/j.bbagrm.2016.08.007 PMC5203810

[btaf210-B57] Tang S, Dunnmon JA, Qu L *et al*. Modeling multivariate biosignals with graph neural networks and structured state space models. *PMLR* 2023;**209**:50–71.

[btaf210-B58] van Deursen JM. The role of senescent cells in ageing. Nature 2014;509:439–46. 24848057 10.1038/nature13193 PMC4214092

[btaf210-B59] Vaswani A, Shazeer N, Parmar N et al Attention is all you need. *Adv Neural Inform Process Syst* 2017;30:5998–6008.

[btaf210-B60] Wang H , Wu Y, Fang R et al Time-Varying gene network analysis of human prefrontal cortex development. Front Genet 2020;11:574543. 33304381 10.3389/fgene.2020.574543 PMC7701309

[btaf210-B61] Wang J , Chen Y, Zou Q. Inferring gene regulatory network from single-cell transcriptomes with graph autoencoder model. PLoS Genet 2023a;19:e1010942. 37703293 10.1371/journal.pgen.1010942 PMC10519590

[btaf210-B62] Wang L , Trasanidis N, Wu T et al Dictys: dynamic gene regulatory network dissects developmental continuum with single-cell multiomics. Nat Methods 2023b;20:1368–78. 37537351 10.1038/s41592-023-01971-3

[btaf210-B63] Wang Y , Joshi T, Zhang X-S et al Inferring gene regulatory networks from multiple microarray datasets. Bioinformatics 2006;22:2413–20. 16864593 10.1093/bioinformatics/btl396

[btaf210-B64] Wang Z , Gerstein M, Snyder M. RNA-Seq: a revolutionary tool for transcriptomics. Nat Rev Genet 2009;10:57–63. 19015660 10.1038/nrg2484 PMC2949280

[btaf210-B65] Wise A , Bar-Joseph Z. cDREM: inferring dynamic combinatorial gene regulation. J Comput Biol 2015;22:324–33. 25844671 10.1089/cmb.2015.0010 PMC4394168

[btaf210-B66] Yan M , Hu J, Yuan H et al Dynamic regulatory networks of T cell trajectory dissect transcriptional control of T cell state transition. Mol Ther Nucleic Acids 2021;26:1115–29. 34786214 10.1016/j.omtn.2021.10.011 PMC8577129

[btaf210-B67] Yosef N , Shalek AK, Gaublomme JT et al Dynamic regulatory network controlling TH17 cell differentiation. Nature 2013;496:461–8. 23467089 10.1038/nature11981 PMC3637864

[btaf210-B68] Zaheer M, Kottur S, Ravanbakhsh S et al Deep sets. Adv Neural Inform Process Syst 2017;30:3391–401.

[btaf210-B69] Zhang B , Upadhyay R, Hao Y et al Multimodal single-cell datasets characterize antigen-specific CD8+ T cells across SARS-CoV-2 vaccination and infection. Nat Immunol 2023;24:1725–34. 37735591 10.1038/s41590-023-01608-9 PMC10522491

[btaf210-B70] Zhu S , Wang Y. Hidden Markov induced dynamic Bayesian network for recovering time evolving gene regulatory networks. Sci Rep 2015;5:17841. 26680653 10.1038/srep17841 PMC4683538

[btaf210-B71] Zhu Y , Xu W, Zhang J et al A survey on graph structure learning: progress and opportunities. arXiv, 2021, preprint: not peer reviewed.

