# Supplementary material for: Recovering time-varying networks from single-cell data
Source: Bioinformatics. 2025 Jul 15;41(Suppl 1):i628–36. doi: 10.1093/bioinformatics/btaf210 (PMC12261490; doi:10.1093/bioinformatics/btaf210)
Supplement: btaf210_Supplementary_Data [file btaf210_supplementary_data.zip › btaf210_Supplementary_Data/Hasanaj.1.alt-text.pdf]

Manuscript: Recovering Time-Varying Networks From Single-Cell Data

Submission No: 1

Figure 1 caption: Overview of Marlene. Marlene takes as input a gene expression matrix that is cell-by-gene. It begins by performing gene featurization using a pooling by multihead attention (PMA) mechanism, producing a gene feature matrix. This matrix is then passed through a self-attention module to generate a gene network in the form of an adjacency matrix. To capture temporal dynamics, the weights of the self-attention module evolve across time points via a gated recurrent unit (GRU). The expression of transcription factors, along with the inferred graph, is then used to reconstruct the full gene expression vector. Finally, the reconstructed matrix is used to predict the cell type of each batch. The model is trained using a model-agnostic meta-learning framework, where each cell type is treated as a "task" to be learned, thus allowing fast adaptation to underrepresented cell types.

Alt text: Flowchart of the Marlene model: gene expression matrix is featurized with PMA, a self-attention module infers a gene network, GRU models temporal dynamics, transcription factor expression and the graph reconstruct gene expression, and the output predicts cell types via meta-learning.

Figure 2 caption: Overlap analysis of the SARS-CoV-2 dataset. Showing — log10 (FDR) values from a Fisher's exact test measuring the overlap between predicted TF-gene interactions in reconstructed networks and two TF-gene interaction databases, TRRUST (top) and RegNetwork (bottom). Each column block is a cell type. Best performing method is starred. Exact values shown in Supp. Fig. 1.

Alt text: Bar plots showing the statistical significance of overlap between predicted transcription factor–gene interactions and two reference databases, TRRUST and RegNetwork, across different cell types.

Figure 3 caption: Temporal analysis of the predicted gene regulatory networks for the SARS-CoV-2 vaccine dataset. (a) Intersection-over-union (IoU) scores between consecutive graphs. (b) For each method, top 3 MSigDB terms enriched for genes that were newly regulated at day 2 but not day 0.

Alt text: Temporal analysis of predicted gene networks for the SARS-CoV-2 vaccine dataset, showing graph similarity over time and top enriched pathways for genes newly regulated at day 2.

Figure 4 caption: Enrichment for senescence using the SenMayo set. For 4 cell types, there was statistically significant enrichment for the oldest age group. We only used the top 200 regulated genes.

Alt text: Senescence enrichment analysis showing four cell types with significant enrichment in the oldest age group.

Table 1 caption: Time series scRNA-seq datasets used in this study. Preprocessing information shown in the supplement.

Alt text: Overview of time series single-cell RNA-seq datasets used in the study.
