# Supplementary material for: Recovering time-varying networks from single-cell data
Source: Bioinformatics. 2025 Jul 15;41(Suppl 1):i628–36. doi: 10.1093/bioinformatics/btaf210 (PMC12261490; doi:10.1093/bioinformatics/btaf210)
Supplement: btaf210_Supplementary_Data [file btaf210_supplementary_data.zip › btaf210_Supplementary_Data/Hasanaj.1.sup.1.pdf]

Supplement: Recovering Time-Varying Networks From Single-Cell Data

1 Set Transformer operations

We redefine the Multihead and rFF operations from Set Transformers [1] to those used for Marlene here.

First, we define the **Attention** operation. Let $Q \in \mathbb{R}^{k\times g}$ be the query matrix of k elements and g dimensions. The Attention operation used for MAB is

$$\text{Attention}(Q, K, V) = \text{softmax}\left(\frac{QK^\top}{\sqrt{g}}\right)V \tag{1}$$

where the key and value matrices are $K, V \in \mathbb{R}^{c\times g}$. Next, the Multihead attention operation with h heads [2] is given by

$$\text{Multihead}(Q, K, V) = \text{concat}(O_1, \ldots, O_h)W^O \tag{2}$$

where $O_j = \text{Attention}(QW_j^Q, KW_j^K, VW_j^V)$ for weight matrices $W_j^Q, W_j^K, W_j^V \in \mathbb{R}^{g\times g/h}$ and $W^O \in \mathbb{R}^{g\times g}$ (these matrices are not to be confused with self-attention weights used consequently for Marlene). In our implementation, k is the number of seeds or output vectors used for the PMA layer. This is a hyperparameter that corresponds to the number of "statistic" vectors we expect to learn from data. Finally, rFF is a feedfoward layer such as a linear layer.

2 EvolveGCN operations

Here, we introduce the GRU and topK pooling operations used in the second step of Marlene.

The topK pooling operation is needed to summarize nodes into k representative ones [3, 4]. Here k is the same as the number of seeds used for PMA. Given an input $\mathbf{G} \in \mathbb{R}^{g\times k}$ and a learnable vector q, the TopK operation performs the following steps:

$$\begin{aligned}
\rho &= \frac{\mathbf{G}q}{\|q\|} \\
i &= \text{Top-k-indices}(\rho) \\
\mathbf{Z} &= [\mathbf{G} \odot \tanh(\rho)]_i.
\end{aligned}$$

At time step t, given a pooled matrix $\mathbf{Z}_t$ and hidden state $\mathbf{W}_{t-1}$ (i.e., self-attention weights $\mathbf{W}_{t-1}^Q$ or $\mathbf{W}_{t-1}^K$), the standard GRU operation is:

$$\begin{aligned}
r_t &= \sigma(M_{ir}\mathbf{Z}_t + b_{ir} + M_{hr}\mathbf{W}_{t-1} + b_{hr}) \\
z_t &= \sigma(M_{iz}\mathbf{Z}_t + b_{iz} + M_{hz}\mathbf{W}_{t-1} + b_{hz}) \\
n_t &= \tanh(M_{in}\mathbf{Z}_t + b_{in} + r_t \odot (M_{hn}\mathbf{W}_{t-1} + b_{hn})) \\
\mathbf{W}_t &= (1 - z_t) \odot n_t + z_t \odot \mathbf{W}_{t-1}
\end{aligned}$$

where σ is the sigmoid function and $\odot$ is the Hadamard product. See also Paszke *et al.* [5].

3 Preprocessing details

The SARS-CoV-2 and HLCA datasets were already preprocessed and log-transformed, so no additional preprocessing was applied. For the mouse dataset, which consisted of raw gene expression values, we followed a standard preprocessing pipeline (e.g., as done in Scanpy [6]): we filtered out cells with fewer than 100 expressed genes and genes expressed in fewer than 100 cells, then applied total-count normalization followed by a log1p transformation.

4 Supplementary figures for the SARS-CoV-2 dataset

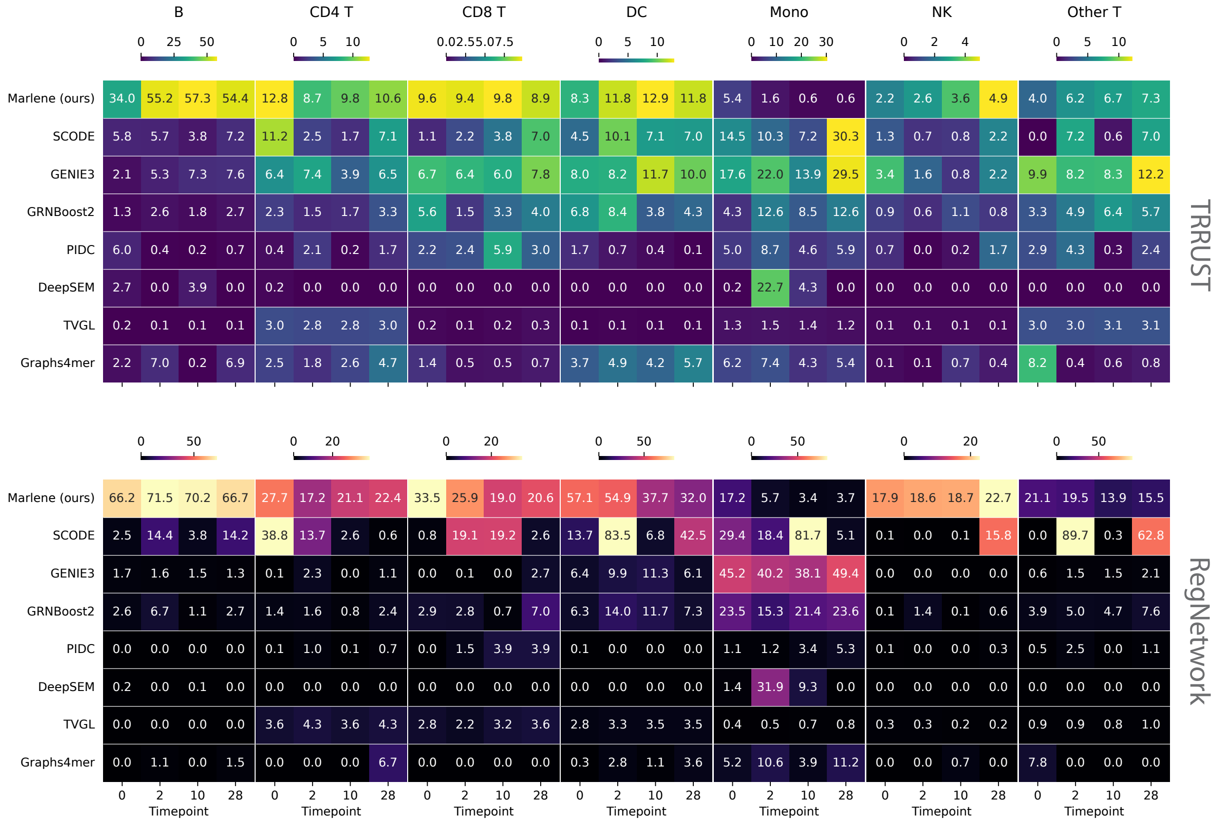

Figure 1: Annotated overlap heatmap similar to Fig. 2.

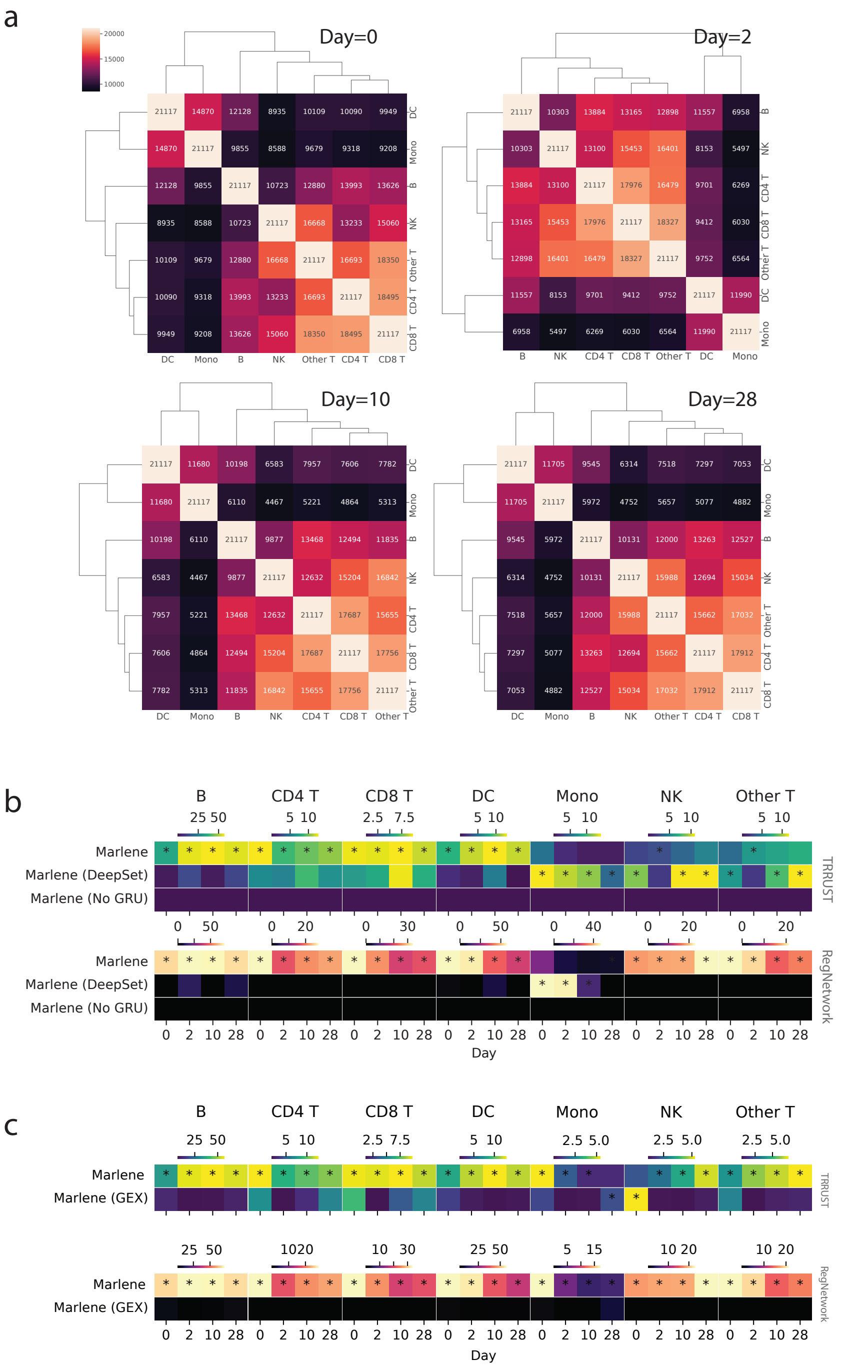

Figure 2: Supplementary results for the SARS-CoV-2 dataset. (a) Pairwise overlap of edge sets across cell types for Marlene at each time point. Only the top 2% edges were considered. (b) Results from the ablation study. For Marlene (DeepSet), the PMA layer was replaced with a DeepSet module. The module consists of two linear layers separated by a ReLU activation function. The first linear layer maps each gene expression value to a vector of size 16. The data is then pooled across cells by summing, followed by a final linear layer to obtain a vector of size 16 for each gene. For Marlene (No GRU), the GRU unit was completely removed, thus leading to independent networks for each time point. (c) Comparison against Marlene trained to reconstruct gene expression (Marlene (GEX)) rather thanc cell types.

5 Supplementary figures for the HLCA dataset

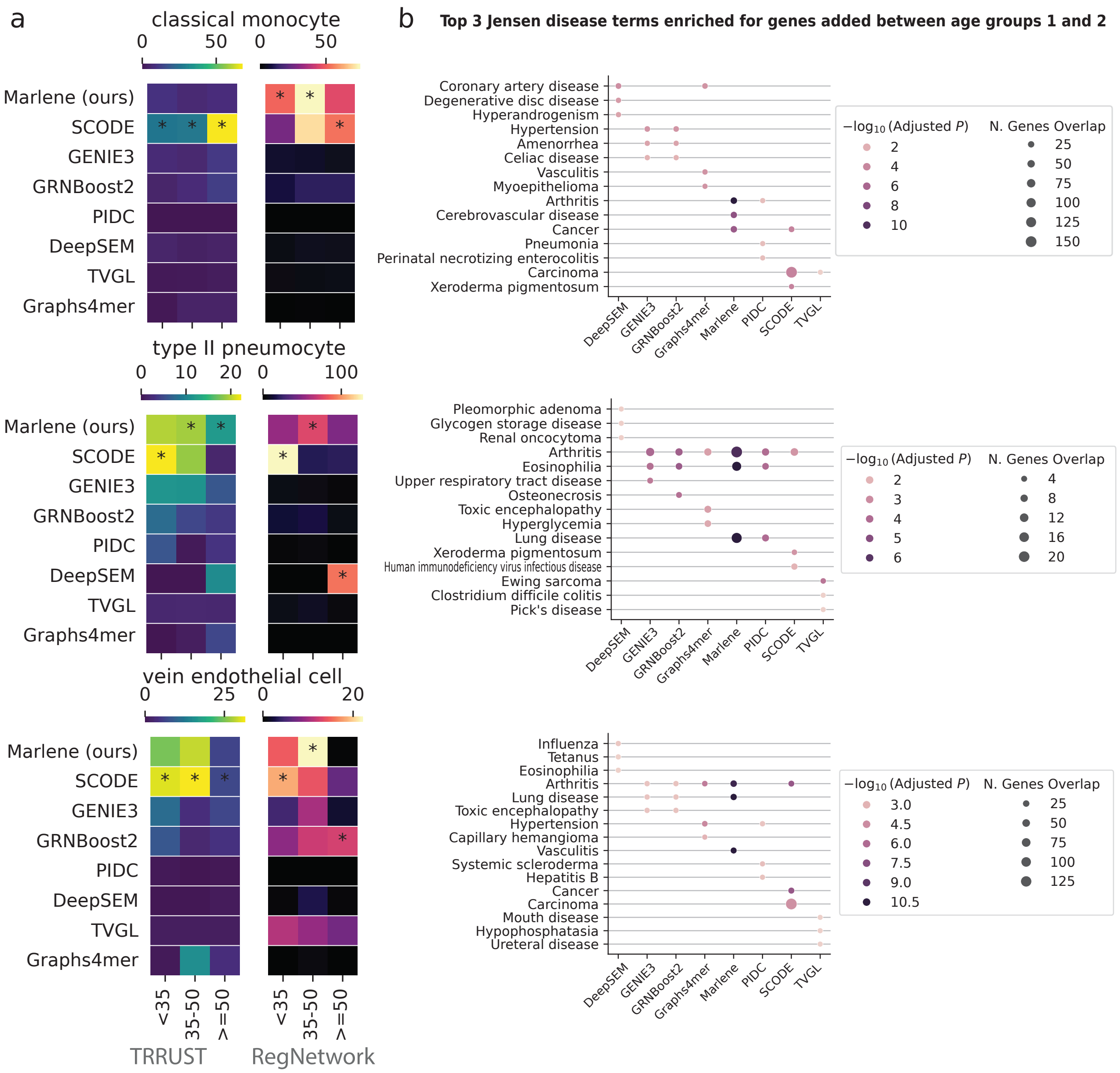

Figure 3: Results on the HLCA dataset. (a) FDR corrected p-values of Fisher exact tests reflecting the number of links that overlap with TRRUST and RegNetwork databases. (b) Top 3 Jensen Diseases terms enriched for genes added between the first and second age group.

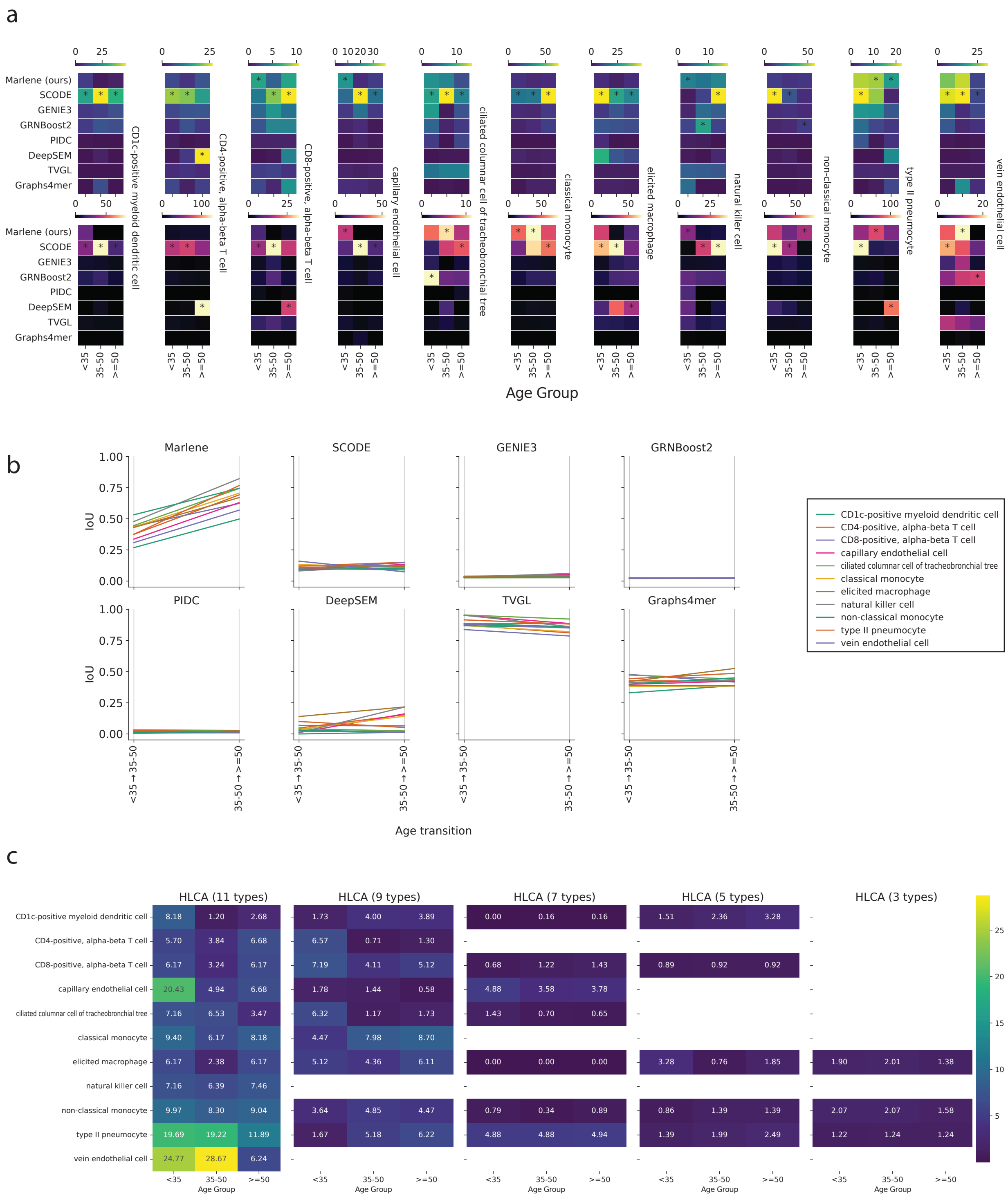

Figure 4: (a) FDR corrected p-values of Fisher exact tests reflecting the number of links that overlap with the two TF-gene databases. (b) IoU scores across time reflecting the overlap between consecutive graphs. (c) Corrected p-values for Marlene while changing the number of cell types. Overall, we observe a decrease in performance as fewer cell types are considered.

6 Supplementary figures for the fibrosis dataset

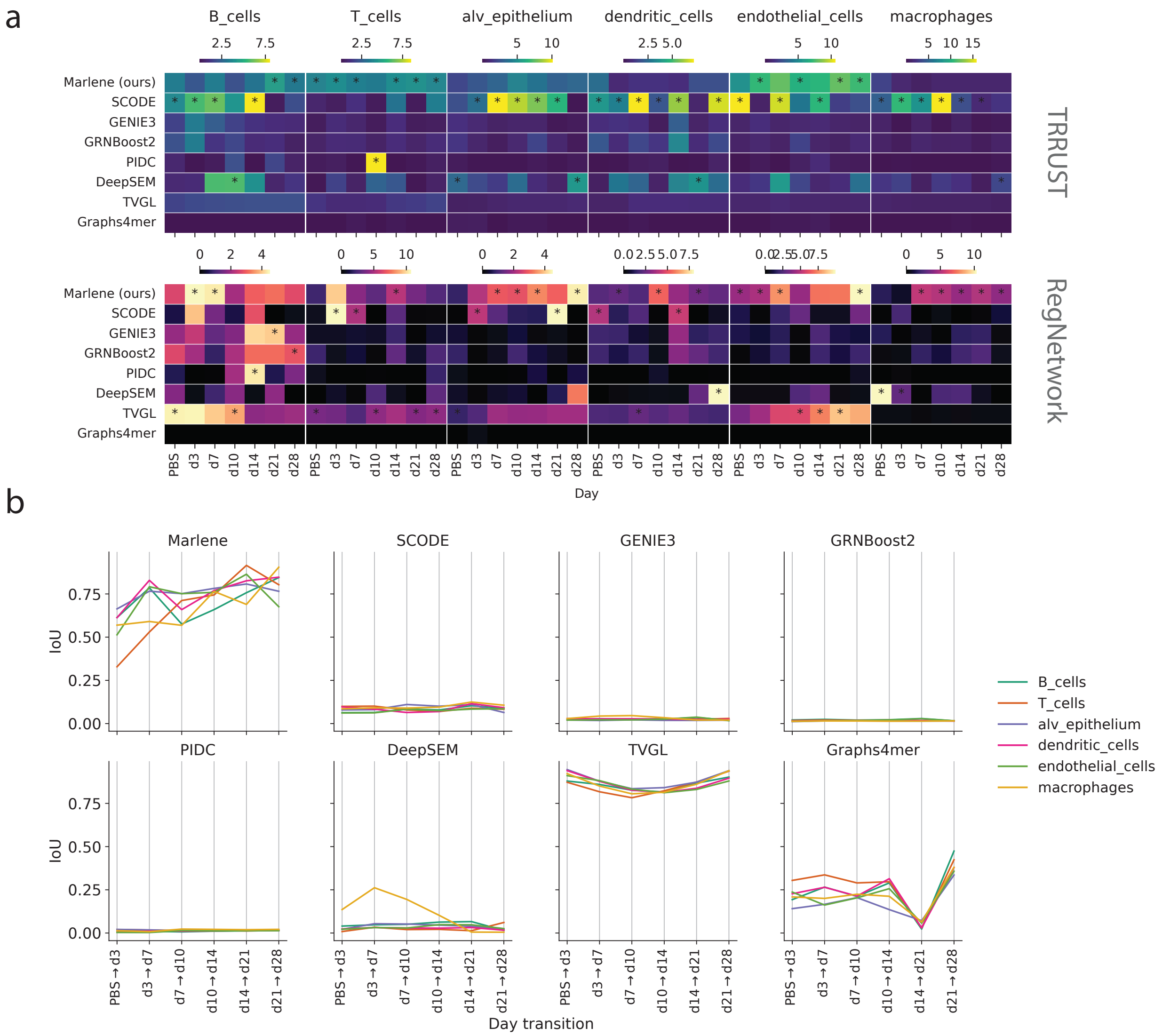

Figure 5: (a) FDR corrected p-values of Fisher exact tests reflecting the number of links that overlap with the two mouse databases. (b) IoU scores across time reflecting the overlap between consecutive graphs.

References

1. Lee, J. *et al. Set Transformer: A Framework for Attention-based Permutation-Invariant Neural Networks* in *Proceedings of the 36th International Conference on Machine Learning* **97** (PMLR, 2019), 3744–3753.
2. Vaswani, A. *et al.* Attention is All you Need. *Advances in Neural Information Processing Systems* **30** (2017).
3. Cangea, C., Veličković, P., Jovanović, N., Kipf, T. & Liò, P. Towards sparse hierarchical graph classifiers. *arXiv [stat.ML]* (Nov. 2018).
4. Pareja, A. *et al.* EvolveGCN: Evolving graph convolutional networks for dynamic graphs. en. *Proc. Conf. AAAI Artif. Intell.* **34,** 5363–5370 (Apr. 2020).

5. Paszke, A. *et al.* PyTorch: An imperative style, high-performance deep learning library. *arXiv [cs.LG]* (Dec. 2019).

6. Wolf, F. A., Angerer, P. & Theis, F. J. SCANPY: large-scale single-cell gene expression data analysis. en. *Genome Biol.* **19,** 15 (Feb. 2018).
